# SIRT1 Inhibition Alleviates Gene Silencing in Fragile X Mental Retardation Syndrome

**DOI:** 10.1371/journal.pgen.1000017

**Published:** 2008-03-14

**Authors:** Rea Biacsi, Daman Kumari, Karen Usdin

**Affiliations:** 1 Section on Genomic Structure and Function, Laboratory of Molecular and Cellular Biology, National Institute of Diabetes, Digestive and Kidney Diseases, National Institutes of Health, Bethesda, Maryland, United States of America; 2 Doctorate School of Biology, Classical and Molecular Genetics Branch, Department of Genetics, Eötvös Lóránd University, Budapest, Hungary; University of Minnesota, United States of America

## Abstract

Expansion of the CGG•CCG-repeat tract in the 5′ UTR of the *FMR1* gene to >200 repeats leads to heterochromatinization of the promoter and gene silencing. This results in Fragile X syndrome (FXS), the most common heritable form of mental retardation. The mechanism of gene silencing is unknown. We report here that a Class III histone deacetylase, SIRT1, plays an important role in this silencing process and show that the inhibition of this enzyme produces significant gene reactivation. This contrasts with the much smaller effect of inhibitors like trichostatin A (TSA) that inhibit Class I, II and IV histone deacetylases. Reactivation of silenced *FMR1* alleles was accompanied by an increase in histone H3 lysine 9 acetylation as well as an increase in the amount of histone H4 that is acetylated at lysine 16 (H4K16) by the histone acetyltransferase, hMOF. DNA methylation, on the other hand, is unaffected. We also demonstrate that deacetylation of H4K16 is a key downstream consequence of DNA methylation. However, since DNA methylation inhibitors require DNA replication in order to be effective, SIRT1 inhibitors may be more useful for *FMR1* gene reactivation in post-mitotic cells like neurons where the effect of the gene silencing is most obvious.

## Author Summary

Fragile X syndrome is the leading cause of heritable intellectual disability. The affected gene, *FMR1*, encodes FMRP, a protein that regulates the synthesis of a number of important neuronal proteins. The causative mutation is an increase in the number of CGG•CCG-repeats found at the beginning of the *FMR1* gene. Alleles with >200 repeats are silenced. The silencing process involves DNA methylation as well as modifications to the histone proteins around which the DNA is wrapped *in vivo*. Treatment with 5-azadeoxycytidine, a DNA methyltransferase inhibitor, reactivates the gene. However, this reagent is toxic and since no DNA demethylase has been found in humans, methylation inhibitors are not useful in cells like neurons that no longer divide. We show here that splitomicin is also able to reactivate the Fragile X allele. It does so by inhibiting a protein deacetylase, SIRT1, thus favoring the action of another enzyme, hMOF that reverses the SIRT1 modification. We also found that 5-azadeoxycytidine acts, at least in part, by reversing the effect of SIRT1. However, since splitomicin reactivation occurred without DNA demethylation, DNA replication is not necessary for its efficacy. Thus, unlike DNA methylation inhibitors, SIRT1 inhibitors may be able to reactivate Fragile X alleles in neurons.

## Introduction

The most common cause of Fragile X mental retardation syndrome (FXS) is the silencing of the *FMR1* gene that occurs when the number of CGG•CCG-repeats in its 5′ untranslated region (5′ UTR) exceeds 200 [1],[2]. The net result is a deficiency in the *FMR1* gene product, FMRP, a protein that regulates the translation of mRNAs important for learning and memory in neurons. How repeats of this length cause silencing is unknown. However, since the sequence of the promoter and open reading frame of these alleles is unchanged, the potential exists to ameliorate the symptoms of FXS by reversing the gene silencing.

The extent of silencing is related to the extent of methylation of the 5′ end of the gene [3],[4],[5]. Treatment of patient cells with 5-aza-dC, a DNA methyltransferase inhibitor, decreases DNA methylation and this is accompanied by partial gene reactivation [4],[5]. However, this compound has 2 major drawbacks: it is extremely toxic and it requires DNA replication to be effective. This would clearly limit its usefulness *in vivo*, particularly in post-mitotic neurons where the FMRP deficiency is most apparent. It also leaves open the question of whether DNA demethylation is necessary for gene reactivation to occur, a situation that for the reasons just mentioned, would severely limit the likelihood that gene reactivation would ever be a viable approach to treating FXS.

While the silenced gene is associated with overall H3 and H4 hypoacetylation, lysine 4 and 9 of histone H3 are the only 2 specific modifiable sites that have been examined thus far. In individuals with FXS, the levels of histone H3 acetylated at K9 (H3K9Ac) and H3 dimethylated at K4 (H3K4Me2) are decreased relative to the normal gene while the level of H3K9 dimethylation (H3K9Me2) is increased [5],[6],[7]. By analogy with other genes that have been studied more extensively, we would expect that there are a number of other histone residues that are differentially methylated or acetylated, when the *FMR1* gene is aberrantly silenced.

The acetylation state of the histones associated with a particular genomic region is thought to play a critical role in regulating gene expression. The level of acetylation is dependent on the dynamic interplay of histone acetyltransferases (HATs) and histone deacetylases (HDACs). HDACs are sometimes divided into 4 functional classes based on sequence similarity. Class I (HDAC1, 2, 3, and 8) and class II (HDAC4, 5, 6, 7, 9, and 10) HDACs remove acetyl groups through zinc-mediated hydrolysis. Class III HDACs, which includes SIRT1, catalyze the deacetylation of acetyl-lysine residues by a mechanism in which NAD^+^ is cleaved and nicotinamide, which acts as an end product inhibitor, is released. Class IV HDACs are HDAC11-related enzymes that are thought to be mechanistically related to the Class I and II HDACs. To date, only inhibitors of Class I, II and IV HDACs have been tested for their ability to reactivate the *FMR1* gene in FXS cells [4],[6],[8]. These HDAC inhibitors (HDIs), which include TSA and short-chain fatty acids like phenylbutyrate, have a much smaller effect on *FMR1* gene reactivation than 5-aza-dC when used alone, although some synergistic effect was noted when these compounds were used in conjunction with 5-aza-dC [5],[6],[7],[9].

Recently, it has become apparent that not only do some HDACs act preferentially on specific lysines on different histones, but they also target certain genes for deacetylation [10]. Thus the available data did not rule out a role for HDACs, specifically Class III HDACs, in gene silencing in FXS. We show here that SIRT1, a member of the Class III HDAC family, plays an important role in silencing of *FMR1* in the cells of Fragile X patients acting downstream of DNA methylation. Furthermore we show that SIRT1 inhibitors result in increased *FMR1* transcription. This increase is associated with an increase in H4K16Ac and H3K9Ac but does not involve DNA demethylation or an increase in H3K4 dimethylation.

## Results

### Inhibitors of NAD^+^-dependent enzymes increase expression of *FMR1* full mutation alleles

**Figure 1:**
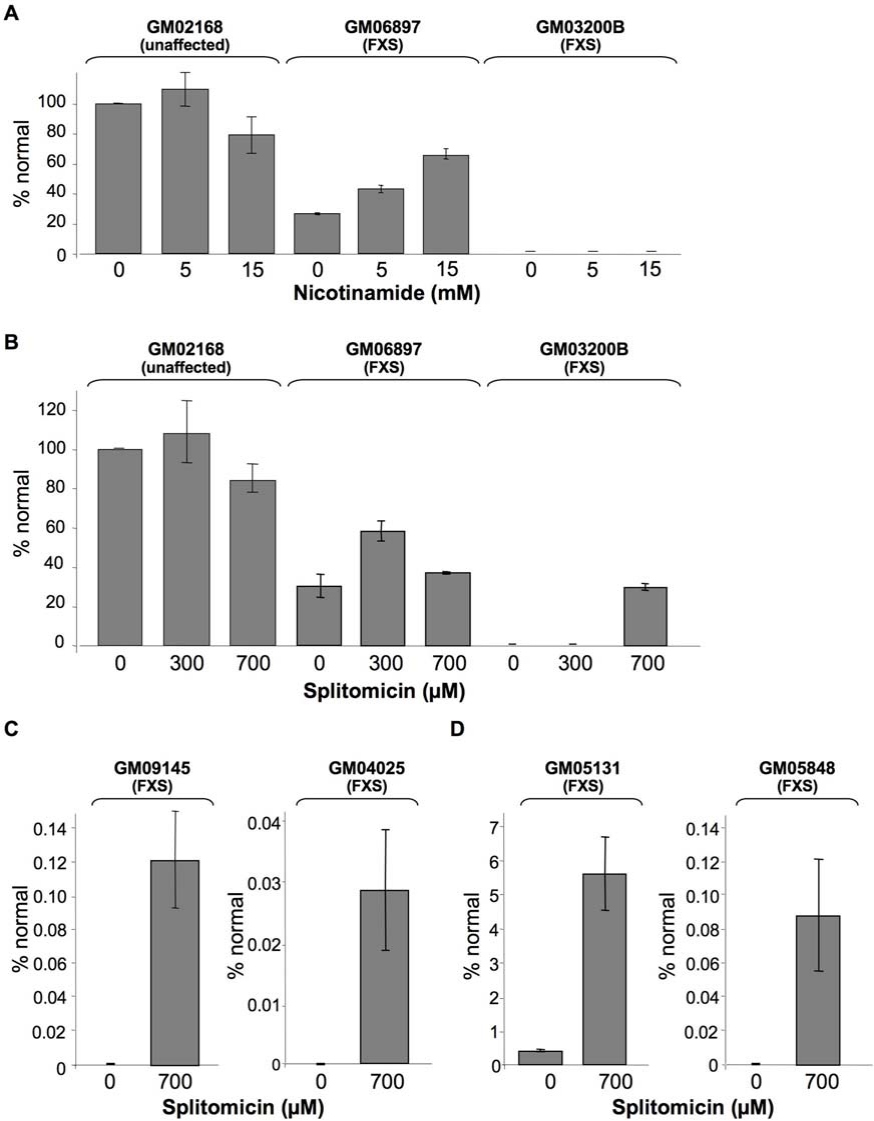
The effect of nicotinamide and splitomicin on *FMR1* gene expression in unaffected and FXS cell lines. (A). Lymphoblastoid cells from an unaffected individual (GM02168), individuals with FXS (GM06897 and GM03200B) treated with the indicated concentrations of nicotinamide. (B and C) Lymphoblastoid cells from an unaffected individual (GM02168), individuals with FXS (GM06897, GM03200B, GM09145 and GM04025) treated with the indicated concentrations of splitomicin. (D) FXS fibroblasts (GM05131 and GM05848) treated with 700 µM splitomicin. *FMR1* mRNA levels were measured by real time PCR using Taqman primer-probe mixes. The *FMR1* expression in patient cells was plotted as a percentage of the *FMR1* mRNA produced from unaffected cells without any treatment. The decrease in *FMR1* mRNA levels at higher nicotinamide and splitomicin concentrations seen in the normal cells (GM02168) was not significant by Students T-test. However, while the effect of 300 µM splitomicin on GM06897 was significant (p = 0.0016), some inhibition of *FMR1* mRNA levels was seen at 700 µM such that *FMR1* mRNA levels were not significantly different in untreated and splitomicin treated cells (p = 0.49). This inhibition was not seen with other cells and may reflect “off-target” effects of splitomicin on other genes/proteins in these cells.

Nicotinamide (Vitamin B3), an end product inhibitor of NAD^+^-dependent enzymes like the Class III HDACs [11], increased *FMR1* expression of a lymphoblastoid cell line from a Fragile X patient with a partially methylated *FMR1* gene (GM06897) [12],[13]. Fifteen millimolar nicotinamide increased *FMR1* mRNA levels by ∼3-fold while having little or no effect on the amount of *FMR1* mRNA produced in normal cells (Figure 1A). A much smaller effect was seen in GM03200B cells in which the *FMR1* gene is more heavily methylated [12],[13] and makes much less *FMR1* mRNA (too small to see on the scale of the graphs shown in Figure 1A).

**Figure 2:**
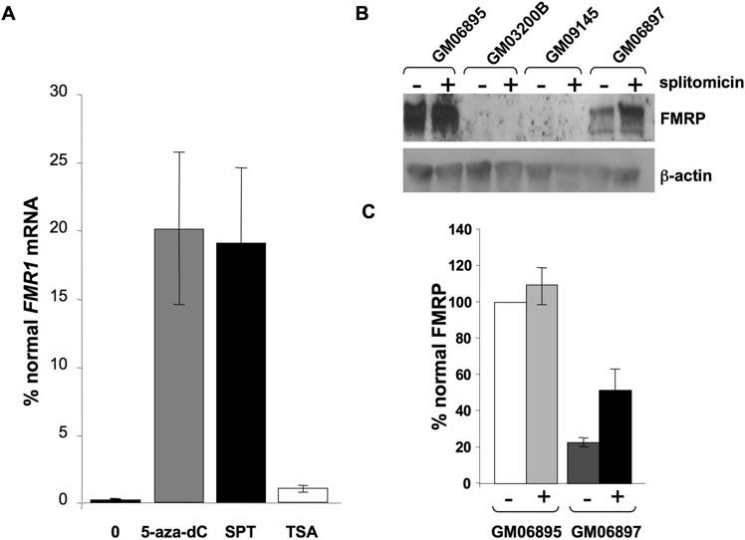
Gene reactivation and FMRP production. (A) The effect of HDAC and DNA methylation inhibitors on *FMR1* gene expression in FXS cells. Lymphoblastoid cells from an unaffected (GM06895) and affected individual (GM03200B) were treated with 10 µM 5-aza-dC for 72 hr, or with 700 µM splitomicin (SPT) or 3 µM TSA for 24 hr. *FMR1* mRNA levels were measured by real time PCR and the *FMR1* expression in patient cells was plotted as a percentage of the *FMR1* mRNA produced from unaffected cells without any treatment. (B) Representative western blot with an anti-FMRP antibody showing the extent of FMRP production in lymphoblasts from unaffected and affected individuals with and without treatment with either 300 µM (GM06897) or 700 µM splitomicin. (C) Quantification of FMRP levels in untreated and splitomicin treated cells. FMRP levels were determined by densitometric analysis. After normalization to β-actin to control for differences in protein loading, the results were expressed as a fraction of the amount of FMRP in untreated cells from an unaffected individual (GM06895). The results shown represent the average of 3 independent experiments. The difference in FMRP levels in GM06897 cells with and without treatment was significant at p = 0.0151.

Splitomicin, a compound with a saturated six-membered lactone ring, is a more specific inhibitor of Class III HDACs and is thought to have a mechanism distinct from that of nicotinamide, inhibiting these enzymes by competing for binding of the acetylated substrate [14]. Splitomicin not only increased *FMR1* mRNA levels in GM06897, but it produced a 200–600-fold increase in the amount of *FMR1* mRNA in cell lines like GM03200B that were only minimally responsive to 15 mM nicotinamide (Figure 1B). This corresponded to a final *FMR1* expression level that was ∼15–25% of normal, depending on which normal cell line was used for comparison. This level of activation was comparable to that achieved with 10 µM 5-aza-dC, an inhibitor of DNA methylation and much higher than the level of activation seen with TSA (Figure 2). The extent of activation was impressive given the low potency of splitomicin (in the micromolar range) and its relative instability (it has a half-life of 30 minutes at neutral pH [14]). A much smaller level of reactivation was seen with GM09145 and GM04025, lymphoblastoid cell lines that are more heavily methylated [12],[13] and that make less *FMR1* than GM03200B (Figure 1C). A similar low level of reactivation was seen for 2 fibroblast cell lines that make very little *FMR1* mRNA in the absence of splitomicin (Figure 1D). The simplest interpretation of these data is that a class III HDAC is involved in downregulating *FMR1* expression from full mutation alleles. As has been reported for 5-aza-dC, the extent of reactivation is inversely related to the extent of silencing [6]. Whether the failure to completely reactivate the *FMR1* gene with either drug reflects a suboptimal dosing strategy or the limits of what these classes of compounds can accomplish remains to be seen.

The ∼2-fold increase in *FMR1* mRNA seen in GM06897 treated with 300 µM splitomicin is accompanied by a ∼2-fold increase in FMRP (Figure 2B and 2C). However, for cell lines where the *FMR1* gene is more heavily methylated and that make no detectable FMRP, splitomicin did not result in the production of detectable levels of the *FMR1* gene product (Figure 2B). The cell lines GM03200B, GM09145 and GM04025 are not only more heavily silenced than GM06897 but they also have more repeats (GM06897 has 477 repeats compared to 530 and 645 for GM03200B and GM04025 respectively). The failure to detect FMRP in these cells may reflect some combination of the low level of gene reactivation with the difficulty translating long CGG-repeat tracts previously reported for lymphocytes and lymphoblastoid cells [15],[16],[17],[18],[19].

### The class III HDAC SIRT1 is involved in the silencing of the *FMR1* gene in FXS cells

**Figure 3:**
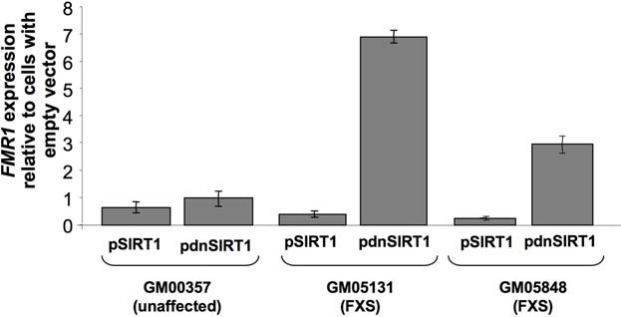
The effect of SIRT1 on *FMR1* gene expression. Vectors expressing either SIRT1 or a dominant negative version of SIRT1 (dnSIRT1) were transfected into fibroblasts from an unaffected individual and individuals with FXS. After 48 hrs *FMR1* mRNA levels were measured by real time PCR and plotted relative to the *FMR1* mRNA produced from cells transfected with empty vector. The results represent the average and standard deviations of 3 independent experiments.

Of the known class III HDACs, only SIRT1 is predominantly nuclear [20]. In order to assess whether SIRT1 was involved in *FMR1* gene silencing, we transfected plasmids encoding a human SIRT1 protein and a dominant negative version of this construct (dnSIRT1) [21] into fibroblast cells from 3 different males, 1 who was unaffected and 2 with FXS. Fibroblasts were chosen because of the relative efficiency of transfection compared to lymphoblastoid cells. Transfection of the FXS fibroblasts (GM05131 and GM05848) with the normal SIRT1 construct led to a decrease in *FMR1* expression from the low level seen in untransfected cells. In contrast a large increase in *FMR1* expression was seen when the dnSIRT1 construct was used (Figure 3). This is consistent with a negative effect of SIRT1 on *FMR1* transcription. Overexpression of these constructs only had a small effect on the level of *FMR1* expression in unaffected individuals analogous to what was seen with nicotinamide and splitomicin.

**Figure 4:**
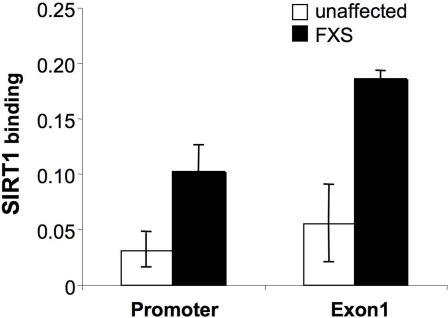
The association of SIRT1 with the *FMR1* promoter in unaffected and affected cells. Fibroblasts were transfected with pCruzWTSIRT1-HA which expresses a SIRT-HA tag fusion protein. ChIP was carried out using anti-HA antibody. Real time PCR was carried out on the immunoprecipitated material and the fold change of the *FMR1* promoter and the first exon DNA were expressed relative to input DNA.

To examine whether the effect of SIRT1 was direct or indirect, we carried out ChIP assays using an anti-HA antibody on a FXS cell line transfected with a construct encoding the HA-tagged SIRT1 [21]. The HA-tagged SIRT1 was enriched on the *FMR1* allele in FXS cells compared to normal alleles (Figure 4).

### Splitomicin increases H4K16 acetylation at the 5′ end of FXS alleles

SIRT1 binding to the promoter would be consistent with a role of this deacetylase in modification of the chromatin associated with the *FMR1* gene in FXS cells. We therefore investigated the chromatin changes caused by splitomicin treatment using ChIP with antibodies to H3K9Ac and H4K16Ac since these are the major residues deacetylated by SIRT1 *in vitro* [22]. We also examined the levels of H3K4Me2, which is a mark of active chromatin that has been shown to increase when FXS alleles are reactivated with 5-aza-dC [7]. We examined the region upstream of the start of transcription and a region of exon 1 downstream of the repeat, with and without, splitomicin treatment. To better understand the differences between gene reactivation mediated by splitomicin and that mediated by 5-aza-dC we also examined the same histone modifications in these cells after 5-aza-dC treatment.

**Figure 5:**
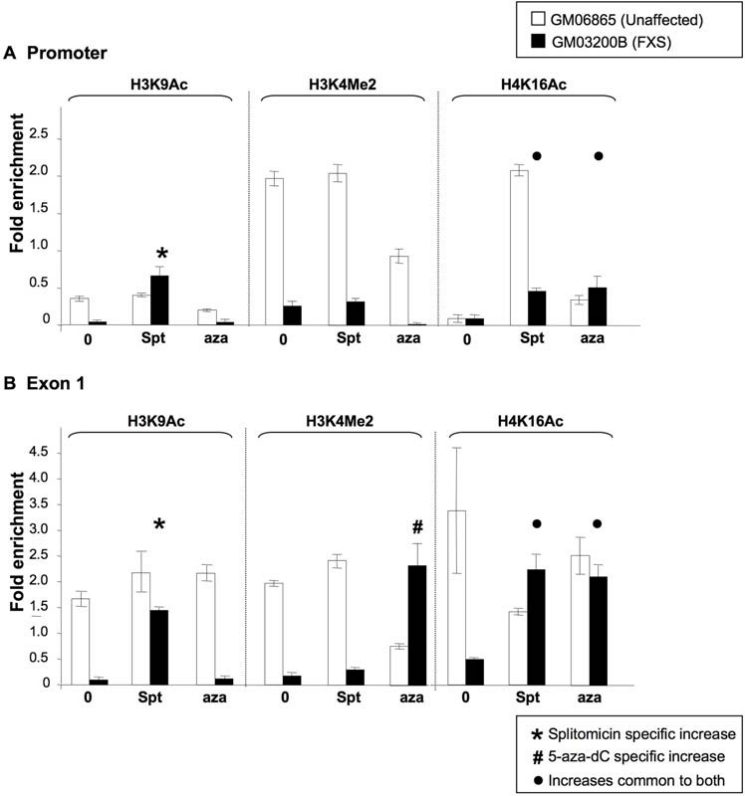
Splitomicin and 5-aza-dC-induced chromatin changes at the 5′ end of the *FMR1* gene in affected and unaffected individuals. Lymphoblastoid cells from an unaffected (GM06865) and affected individual (GM03200B) were treated with 700 µM splitomicin and 10 µM 5-aza-dC as before. ChIP was performed using antibodies to H3K9Ac, H4K16Ac and H4Kme2. Real time PCR was carried out on the immunoprecipitated material and the results expressed as the percentage of input DNA and normalized to GAPDH. Panels A depicts the chromatin modifications occurring in untreated and treated cells in the promoter region. Panel B depicts the chromatin modifications occurring in untreated and treated cells in exon 1.

Both the promoter and exon 1 from a normal allele had higher levels of H3K9Ac and H3K4Me2 than the heavily silenced *FMR1* full mutation allele, consistent with previous reports (Figure 5A and 5B, left and center panels). In unaffected cells splitomicin had little, if any, effect on the level of H3K9Ac on either the promoter or exon 1 (Figure 5A and 5B, left panel). However, splitomicin treatment of FXS cells increased H3K9Ac on ∼2-fold on the promoter and on ∼15-fold on exon 1. The net result of this increase is that H3K9Ac levels in FXS cells treated with splitomicin are very similar to that seen in normal cells. This suggests that SIRT1 is responsible for the hypoacetylation of H3K9 seen on FXS alleles, consistent with the observed *in vitro* properties of SIRT1 [22]. In contrast, 5-aza-dC had no effect on H3K9Ac in this region. The opposite situation was seen with H3K4Me2, in that splitomicin had no effect while 5-aza-dC caused a large increase in H3K4Me2 levels on exon 1 of the FXS allele (Figure 5B, center panel). However, both splitomicin and 5-aza-dC increased the levels of H4K16Ac on both the promoter and exon 1 of the FXS allele (Figure 5A and 5B, right panel). This suggests that DNA methylation and SIRT1 may act in the same or overlapping pathways and that this modification may play a key role in *FMR1* gene silencing.

**Figure 6:**
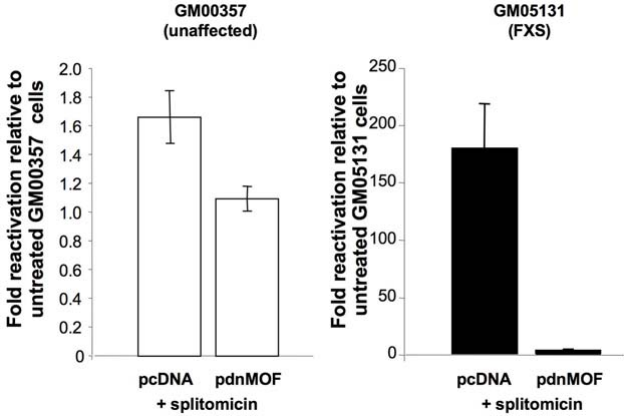
The effect of hMOF on splitomicin-mediated *FMR1* gene reactivation. Fibroblasts from affected and unaffected individuals were treated with 700 µM splitomicin after being transfected with either empty pcDNA3 vector or with the vector containing a dominant negative version of hMOF. The *FMR1* expression was measured by real time PCR and expressed as the fold change relative to the levels of *FMR1* seen in cells without splitomicin treatment.

To assess the contribution of H4K16 acetylation to splitomicin-mediated *FMR1* gene reactivation, we examined the effect of hMOF, a histone acetyltransferase that specifically targets H4K16 [23], on splitomicin-treated patient cells. As can be seen in Figure 6, transfection of patient fibroblasts with a dominant negative version of hMOF completely blocked the splitomicin-mediated increase in *FMR1* mRNA, confirming the importance of H4K16 acetylation in *FMR1* gene reactivation.

### Splitomicin-mediated gene reactivation occurs without significant DNA demethylation

**Figure 7:**
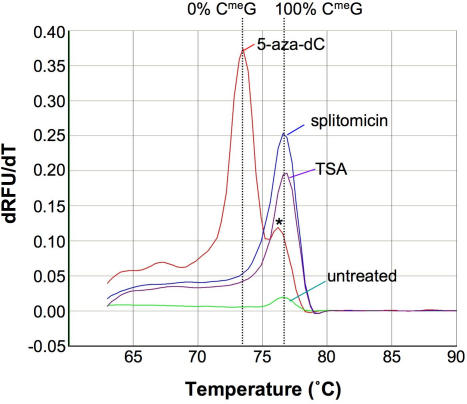
The effect of treatment with 5-aza-dC, splitomicin and TSA on the DNA methylation of the promoter of a FXS allele. Lymphoblastoid cells from a FXS patient were treated with the indicated compounds as described in the Materials and Methods. Genomic DNA isolated from cells with and without treatment was tested for DNA methylation at the *FMR1* promoter. The derivative of the dissociation curve of the bisulfite modified PCR fragment obtained from this procedure (dRFU/dT) was plotted as a function of temperature. The point of inflection corresponds to the Tm of the PCR product. Note that 2 peaks in the 5-aza-dC-treated samples are seen, one corresponding to completely demethylated alleles and a much smaller one, indicated by an asterisk, reflecting residual partially methylated alleles. RFU: relative fluorescent units.

To examine the contribution of DNA demethylation to splitomicin-mediated gene reactivation we used an assay that monitors a region containing 8 CpG residues that is located just upstream of the CGG•CCG-repeat in the *FMR1* gene [24]. Demethylation of a single cytosine produces a 0.5°C drop in the Tm of the PCR product obtained after bisulfite treatment. Reactivation with splitomicin did not change the Tm of the PCR product (Figure 7), suggesting that little, if any, demethylation occurred in this region. DNA demethylation-independent gene reactivation by splitomicin has also been seen in certain tumor suppressor genes aberrantly silenced in cancer cells [25].

In contrast, when these cells are treated with 5-aza-dC the Tm of the PCR product was indistinguishable from the results obtained from unaffected individuals (Figure 7). This is consistent with previous reports of the almost complete demethylation of the promoter by this treatment [4],[6],[9],[26].

## Discussion

We have shown that SIRT1, a class III HDAC, is involved in repeat-mediated *FMR1* gene silencing via the deacetylation of H3K9 and H4K16. Our data suggests that deacetylation of H4K16 is also one of the major downstream consequences of DNA methylation. Since SIRT1 inhibition is able to reactivate the gene without affecting DNA demethylation, DNA methylation is not dominant over chromatin modifications like H4K16Ac with regard to gene expression. Furthermore, it demonstrates that DNA demethylation is not necessary for relieving gene silencing. This resembles the situation in Friedreich ataxia, another Repeat Expansion Disease, in which expanded alleles that are also aberrantly methylated at the DNA level [27], can be reactivated using an HDI alone [28].

**Figure 8:**
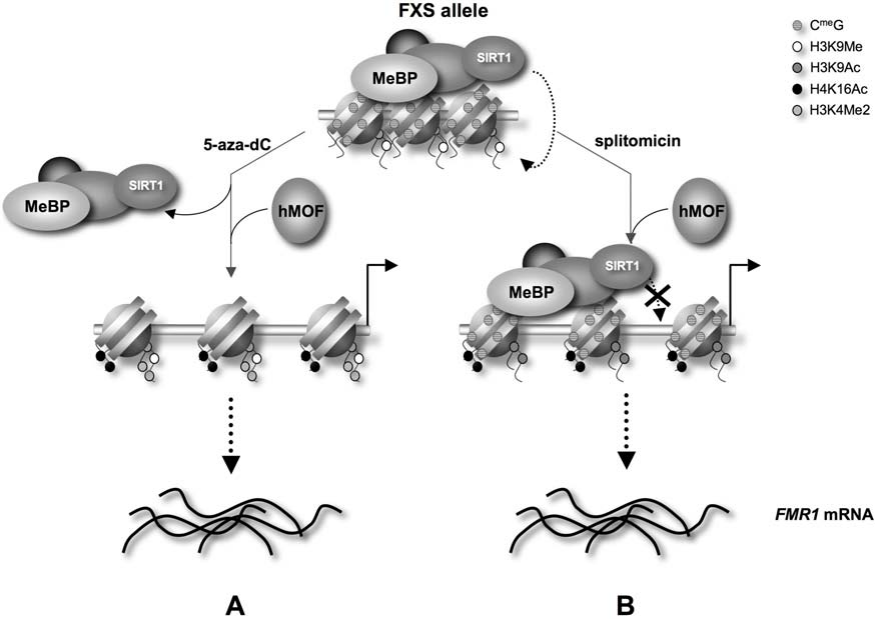
Model for the effect of 5-aza-dC and splitomicin on reactivation of *FMR1* full mutation alleles. Binding of a DNA methyl-binding protein (MeBP) to the methylated 5′ end allows SIRT1 to be recruited. This results in deacetylation of H4K16 and H3K9. The deacetylated H3K9 can now be methylated. A) Inhibition of DNA methylation prevents binding of the MeBP and thus the recruitment of SIRT1. This facilitates the acetylation of H4K16 by hMOF, which promotes chromatin opening and transcriptional activation. B) Inhibition of SIRT1, allows hMOF to acetylate H3K16 and thus to adopt a more open chromatin conformation without affecting DNA methylation.

The increased acetylation of H4K16 seen after treatment with both 5-aza-dC and splitomicin is important since the H4K16 acetylation status is thought to be a key determinant of chromatin accessibility [29]. However, the outcomes of the 2 treatments are not completely equivalent. DNA demethylation by 5-aza-dC is accompanied by an increase in H3K4Me2 that is not seen with splitomicin treatment. In contrast, splitomicin, but not 5-aza-dC, causes acetylation of H3K9. One interpretation of our data is that silenced alleles are associated with a methyl-binding protein or protein complex (MeBP) that binds to the methylated promoter and recruits SIRT1 (Figure 8). SIRT1 in turn deacetylates H3K9, H4K16 and potentially other residues as well. DNA demethylation causes the dissociation of the MeBP-SIRT1 complex from the promoter and creates conditions that favor the recruitment of H3K4 methylases and hMOF which specifically acetylates H4K16, but does not facilitate recruitment of a HAT that uses H3K9 as a substrate (Figure 8A). Splitomicin treatment, on the other hand, inhibits SIRT1 while leaving the promoter methylated. This helps generate a chromatin context conducive to recruiting both hMOF and an H3K9 HAT, but not an H3K4 methyltransferase (Figure 8B). Despite the differences in the final histone modification profile, the extent of gene reactivation resulting from the use of these compounds is similar and they show little additive effect when used in combination (data not shown). This raises the possibility that the most significant action of both compounds is exerted via the acetylation of H4K16 with both H3K4Me2 and H3K9Ac having little direct effect on gene expression.

Since the effect of splitomicin is not dependent on DNA replication, SIRT1 inhibitors may be more useful than 5-aza-dC for reversing *FMR1* gene silencing in neurons which no longer divide and where the absence of FMRP is most debilitating. However, there are significant barriers to using SIRT1 inhibitors to treat FXS. Firstly, Sir2p, the yeast homolog of SIRT1, plays a role in the extension of lifespan in yeast [30] raising the possibility that SIRT1 inhibition may reduce lifespan in humans. However, there is some evidence that SIRT1 actually limits lifespan in mammals, at least in response to chronic genotoxic stress [31]. Furthermore, SIRT1 inhibition sensitizes cancer cells to apoptosis while sparing normal cells, making HDAC III inhibitors promising anti-cancer drugs [32]. It could also be argued that inhibition of HDACs could lead to inappropriate expression of other genes, which could be deleterious. However several HDIs are already approved for use in humans including dihydrocoumarin, an FDA approved food additive and valproate, a broad spectrum HDI, that has been used for decades in the treatment of epilepsy and is also an effective mood stabilizer. Today Valproate is one of the most highly prescribed antiepileptic drugs [33] and is already used in Fragile X patients to treat seizures, aggression and depression [34].

The fact that RNA with long CGG-repeat tracts is thought to be responsible for the Fragile X associated tremor and ataxia syndrome, a late onset neurodegenerative disorder seen in carriers of *FMR1* premutation alleles [35], is a more general problem applicable to any gene reactivation approach for treating FXS. However, some HDIs have actually been shown to be neuroprotective [36],[37] and to expedite the recovery of learning and memory lost as a result of induced neurodegeneration [38]. Thus the beneficial effects of HDIs may help offset the negative effect of the expression of long CGG-repeat tracts.

The final impediment to gene reactivation approaches is the difficulty translating *FMR1* transcripts with long CGG-tracts that has been seen in cells like lymphocytes and lymphoblasts [15],[16],[17],[18],[19]. However, there is reason to think that the translation difficulties do not affect all cells equally. For example, in Fragile X embryonic stem cells where the repeat is still unmethylated, both *FMR1* mRNA and FMRP are made [39]. Furthermore we have shown that the negative effect of the repeats on translation is more severe in some parts of the mouse brain than others [16]. This is consistent with the fact that individuals with unmethylated full mutations show only mild symptoms of FXS [40],[41],[42]. It could thus be argued that when the *FMR1* gene is not silenced, translation occurs at adequate levels in those parts of the brain critical for learning and memory. Even in lymphocytes and lymphoblastoid cells with ∼400 repeats some FMRP is made without treatment ([43] and this manuscript). The fact that even the GM06897 lymphoblastoid cell line, which has 477 repeats, makes some residual FMRP and that FMRP levels increase when the cells are treated with splitomicin, raises the possibility that increased RNA production may lead to increased FMRP production in the ∼40% of individuals with FXS who have repeats of <500 (Sally Nolin, personal communication). Even in lymphoblastoid cells there have been reports of FMRP production in cell lines with >800 repeats after reactivation with 5-aza-dC [4]. New SIRT1 inhibitors with higher stability, selectivity or potency [44] may allow the level of *FMR1* transcription from previously silenced alleles to approach that seen in carriers of unsilenced full mutations. Since HDIs do not require DNA replication to be effective, this class of compounds may thus have therapeutic potential at least in that subset of individuals with repeat numbers that do not preclude translation.

## Materials and Methods

### Cell lines, Plasmids and Reagents

Lymphoblastoid cells (GM02168, GM06895) and fibroblasts (GM00357) from unaffected males and lymphoblastoid cells (GM03200B, GM04025, GM09145) and fibroblasts (GM05131 and GM05848) from males with FXS were obtained from the Coriell Cell Repository (Camden, NJ). The antibodies used in this study were obtained from the following sources: anti-acetyl-Histone H4 (Lys 16) (Cat. #: ab1762) and anti-HA-tag (Cat. #: ab9110) were purchased from Abcam (Cambridge, MA); anti-acetyl-Histone H3 (Lys9) (Cat. #: 07-352), anti-dimethyl-Histone H3 (Lys 4) (Cat. #: 07-030) and anti-rabbit Ig were purchased from Millipore (Temecula, CA). Splitomicin and TSA were obtained from Tocris (Ellisville, MO). Nicotinamide and 5-aza-dC were obtained from Sigma (St. Louis, MO). The mutant human MOF (hMOF) construct in pcDNA3 was a kind gift of Arun Gupta (Washington University School of Medicine, St. Louis, MO). The pCRUZ-HA vector, pCRUZ-HA-SIRT1 and a dominant negative version of this construct were kindly provided by Toren Finkel (NHLBI, NIH, Bethesda, MD).

### Cell culture

Lymphoblastoid cells were cultured in RPMI medium supplemented with 10% fetal bovine serum and 100 units each of penicillin and streptomycin (Invitrogen, Gaithersburg, MD). Fibroblasts were cultured in Minimum Essential Medium supplemented with 1% Glutamax, 10% fetal bovine serum and 100 units of penicillin and streptomycin (Invitrogen). All cells were grown at 37°C in 5% CO2. Cells were treated where indicated with either 300 µM or 700 µM splitomicin, 15 mM nicotinamide, or 3 µM TSA for 24 hours or 10 µM 5-aza-dC for 72 hours. Transfection of fibroblasts was carried out using Fugene 6 (Roche USA, Nutley, NJ) according to the supplier's instructions.

### Analysis of RNA expression levels

Total RNA was isolated from the cell lines using Trizol (Invitrogen) and reverse transcribed using SuperScript™ III RT First Strand Synthesis system for RT-PCR (Invitrogen), as per the manufacturer's instructions. Real time PCR was carried out using an ABI 7500 FAST PCR system (Applied Biosystems, Foster City, CA) using TaqMan™ Universal PCR master mix and *FMR1* and *GUS* Taqman probe primer mixes (Applied Biosystems). For quantitation the comparative threshold (Ct) method was used with normalizing to GUS. The fold change was calculated by comparing the normalized treated *versus* untreated Ct values.

### Chromatin immunoprecipitation assays

The ChIP assay kit from Upstate was used according to the manufacturer's instructions with slight modifications as previously described [27]. The amount of *FMR1* promoter and exon 1 DNA immunoprecipitated with each antibody was determined using quantitative real time PCR as described below. Real time PCR was carried out using an ABI 7500 FAST PCR system and the Power SYBR™ Green PCR kit (Applied Biosystems). For amplification of the promoter region Promoter-F (5′-ACAGTGGAATGTAAAGGGTTG-3′) and Promoter-R (5′-GTGTTAAGCACTTGAGGTTCAT-3′) were used. This primer pair amplifies the 140 bp region from 146800256–146800396 of the human genome sequence (March, 2006 assembly, http://genome.ucsc.edu/cgi-bin/hgBlat) which terminates 736 bp upstream of the 3′ most transcription start site. For amplification of exon 1, the primer pair Exon1-F (5′-CGCTAGCAGGGCTGAAGAGAA-3′) and Exon1-R (5′-GTACCTTGTAGAAAGCGCCATTGGAG-3′) was used. This primer pair amplifies the region 146801368–146801444 of the human genome sequence that corresponds to the region in exon 1 236–311 bp downstream of the transcription start site. All experiments were done in triplicate. The ChIP experiments were performed in triplicate and each PCR reaction was done in duplicate. The immunoprecipitated DNA was expressed relative to the amount of input DNA that constituted 10% of the original material. GAPDH was used for normalization using *hs*_GAPDH exon1F1 primer (5′-TCGACAGTCAGCCGCATCT-3′) and *hs*_GAPDH intron1R1 (5′-CTAGCCTCCCGGGTTTCTCT-3′).

### DNA methylation analysis

Genomic DNA from cell lines was bisulphite modified according to standard procedures except that the bisulphite treatment was carried out overnight at 55°C. The methylation status of the promoter was determined as previously described [24].

### FMRP analysis

SDS protein gel electrophoresis and Western blotting of protein extracts was carried out using standard procedures. Anti-FMRP antibody (MAB2160, Millipore) was used to detect FMRP. Anti-β-actin antibody (Abcam) was used to normalize the FMRP levels for variations in protein loading. Detection of antibody binding was carried using an ECL™ kit (Amersham, Buckinghamshire, UK) according to the manufacturer's instructions. The amount of FMRP and β-actin were determined by standard densitometry. The increase in FMRP was calculated based on the average of 3 independent experiments.
